# An Ecological Approach to Control Pathogens of *Lycopersicon esculentum* L. by Slow Release of Mancozeb from Biopolymeric Conjugated Nanoparticles

**DOI:** 10.3390/jox12040023

**Published:** 2022-11-09

**Authors:** Ravinder Kumar, Vikash Nain, Joginder Singh Duhan

**Affiliations:** 1 Department of Biotechnology, Chaudhary Devi Lal University, Sirsa 125055, India; 2 Department of Food Science and Technology, Chaudhary Devi Lal University, Sirsa 125055, India

**Keywords:** chitosan–gum acacia nanocomposite, tomato pathogens, slow-release of mancozeb, eco-friendly, biopolymers

## Abstract

To control insects, weeds, and infections in crops, old-fashioned pesticide formulations (with massive quantities of heavy metals and a variety of chemicals) are used. By biological amplification via the food chain, many of these established pesticide formulations have accumulated in living systems and caused environmental pollution. To form a nanoparticulate matrix with a diameter ranging from 322.2 ± 0.9 to 403.7 ± 0.7 nm, mancozeb was embedded in chitosan–gum acacia (CSGA) biopolymers and loadings were confirmed via TEM and FTIR. Differential scanning calorimetry analyses were carried out as part of the investigation. Inhibition of *Alternaria alternata* by nanoparticles (NPs) with 1.0 mg/mL mancozeb (CSGA-1.0) was 85.2 ± 0.7 % at 0.5 ppm, whereas for *Stemphylium lycopersici* it was 62.1 ± 0.7% in the mycelium inhibition method. NPs demonstrated antimicrobial action in pot house environments. After ten hours, the mancozeb was liberated from the nanoformulations due to polymer matrix diffusion and relaxation, compared to 2 h for commercial mancozeb. Even while drug-loaded conjugated nanoparticles have equivalent antifungal activities, they have a lower release rate and, hence, reduced toxicology compared to commercial mancozeb. Therefore, this method can be employed to implement sustainable farming techniques in the future.

## 1. Introduction

Old-fashioned pesticide preparations involve enormous amounts of heavy metals and diverse chemicals that are used to control agricultural insects, weeds, and pathogens. Conversely, many of these established pesticide formulations have mounted up in living systems through biological magnification via the food chain. Thus, they also contaminate soil and water atmospheres, harm living entities, and cause disturbances in the equilibrium of the ecological unit [1]. Approximately 90% of applied agrochemicals are lost as run-off during the application, affecting both the environment and farmers’ application costs [2]. Pest resistance upsurges due to indiscriminate pesticide use, which reduces soil biodiversity, kills beneficial soil bacteria, causes biomagnification pollinator decreases, and eliminates the bird’s natural habitat [3]. Tomato (*Lycopersicon esculentum*) is a major worldwide crop that contributed over IND 218 billion to the Indian economy in 2018. The tomato crop is susceptible to various fungal infections throughout pre-harvest and post-harvest periods (https://www.statista.com/statistics/1080566/india-economiccontribution-of-tomatoes/, accessed on 7 September 2022). Primary pathogenic fungi that impact tomato plant growth and development are mainly *A. alternata* and *S. lycopersici*. Mancozeb, a broad-spectrum contact fungicide, has shown strong fungicidal activity in various horticultural crops and accounted for 20% of the global fungicide market (Fungicides Market, 2017–2025 (https://www.prnewswire.com, accessed on 7 September 2022). The fungicide disrupts lipid metabolism and respiration by acting on the sulfhydryl groups of amino acids and enzymes in fungal cells. It is easily water-soluble and can pollute water bodies; its stability is affected by conditions, including light, temperature, humidity, and pH [4]. The maximum residue limit for mancozeb (dithiocarbamates) in living matter is 0.01–25 ppm [5], yet residue levels higher than this have been found in tomatoes [6].

These flaws can be mitigated by encasing the chemical fungicide in a polymer casing, such as chitosan or gum acacia, for long-term release. They are important for encapsulating active ingredients because of their biocompatibility and capacity to encapsulate molecules with diverse physicochemical properties [7]. Chitosan is a natural biodegradable polymer made from the deacetylation of chitin (found naturally in crustaceans, insects, and mollusks) and has a wide range of antifungal, antibacterial, and medicinal uses. It has been recognized as a plant growth stimulant [8]. Chitosan and its nanoparticles (NPs) can also control infections and prevent crop pathogens in cereals and horticulture crops by activating defense-related enzymes [9,10,11]. Gum acacia is a natural gum made from the hardened sap of acacia species. It is a complex mixture of glycoproteins and polysaccharides, with arabinose and galactose as the primary sugars. Because of its viscous nature, it is a non-ionic, non-toxic, and biocompatible bio-polymer utilized in drug delivery [11].

Nanomaterials are synthesized using various entities, manipulated, and utilized to treat various plant ailments [12,13,14]. According to researchers, microorganisms have been identified as potential eco-friendly nano-factories for managing plant diseases [15,16,17,18]. Nanotechnology balances minimal concentrations, maximum pest control, and safe concentrations, resulting in lowering pest management costs [19]. NPs have large surface area to volume ratios and are easily absorbed. Compared to parent formulations, nano-encapsulation technology has been used for commercial pesticides, promising increased potency in precise discharges, targeted deliveries, and environmental and physical stability [20,21]. The generation of reactive oxygen species (ROS), which causes oxidative stress and cell death, is thought to be linked to the antimicrobial effects of NPs [22].

This study described an eco-friendly alternative to using harmful chemicals (commercial mancozeb fungicide) to control phytopathogenic tomato fungi and simultaneously help control environmental pollution and soil health. This may be due to the sustained-release behavior of fungicides from biopolymeric chitosan–gum acacia nanocomposites and presents advanced knowledge of the existing methods and techniques of pesticide applications with reduced leaching.

## 2. Materials and Methods

### 2.1. Reagents and Materials Used

Sigma-Aldrich (St. Louis, MO, USA) provided the chitosan (deacetylation 75%) and sodium tripolyphosphate (TPP). Qualigen, India, supplied gum acacia. Fungicide mancozeb was acquired locally. Hi-Media, Ltd., Mumbai, provided the dialysis tubing. Tomato seeds (Arun Hisar) were bought from the state agricultural university’s vegetable section. A polyhouse was used to conduct an in vivo pot experiment. The Vero cell line was kept at the National Research Centre on Equines, Hisar. 

ITCC, New Delhi, provided the pathogenic fungi (*Alternaria alternata* and *Stemphylium lycopersici*). The cultures were resurrected according to the manufacturer’s instructions.

### 2.2. Synthesis of Blank and Mancozeb Loaded Chitosan–Gum Acacia Conjugated Nanoparticles

Ionic gelation and polyelectrolyte complexation were used to make conjugated nanoparticles. Liquidizing 1.0 mg/mL of chitosan in 1.0% glacial acetic acid (*v/v*) and stirring overnight to dissolve entirely yielded a stock of chitosan at a native pH. Different amounts of solid mancozeb were introduced in three conical flasks of 100 mL capacities, along with a 20 mL stock of chitosan in each flask. Adding a stock solution of aqueous gum acacia (1.0 mg/mL, native pH) to each of the above flasks, dropwise, resulted in final concentrations of mancozeb of 0.5, 1.0, and 1.5 mg/mL, respectively. Magnetic stirring continued for another ten minutes. After that, dropwise, we added 2.0 mL of 1.0% TPP to the solution and stirred for 45 min. Each flask received 10 µL of Tween-20; we stirred the flasks for another 30 min. The suspension was centrifuged at 12,000 rpm for 20 min; the pellet was rinsed with 10 mL of double-distilled water (DDW), and the pellet was kept at 4 °C in a 1.5 mL centrifuge vial for future investigation.

Except for the inclusion of mancozeb, all of the previous processes were followed to synthesize the blank NPs.

### 2.3. Optimization of Experimental Design

Design-Expert Software (Version 8.0.4, Stat-Ease, Inc., MN, USA) was used to optimize the experiment. Mancozeb-loaded chitosan–gum acacia NPs were optimized using a standard protocol via a central composite design. Three factors, i.e., concentrations of chitosan, gum acacia, and mancozeb, varied, and TPP concentration was constantly retained. The particle size was chosen as the response variable.

### 2.4. Characterization

#### 2.4.1. Size, Polydispersity Index, and Zeta Potential

The synthesized conjugated NPs were characterized by a particle size analyzer (PSA) using Zetasizer Nano ZS90 (Malvern Instrumentations, Malvern, UK). Moreover, 50 µL NPs were dispensed in a cuvette and disseminated in a 950 µL DDW to measure the percentage intensity at 25 °C.

#### 2.4.2. Fourier Transform Infrared (FTIR) Spectroscopy

At 80 °C under 0.4 bar, the particles were freeze-dried using a freeze-dryer (Christ, Germany). As a cryoprotectant, mannitol (10%) was utilized. With a fine powder of 5.0 mg, Fourier transform infrared (FTIR) spectroscopy was accomplished. At an average temperature between 4000 and 400 cm^−1^, FTIR spectra were documented using potassium bromide (KBr) in a 1:10 ratio with an AVATAR 370 FTIR (Therma Nicolet, San Jose, CA, USA). A graph pattern was employed to determine the ionic interaction between samples to measure mancozeb loading. Spectroscopic Tools, 2019 (Thomas St. (http://www.science-and-fun.de/tools/, accessed on 7 September 2022) was used to evaluate the data.

#### 2.4.3. Transmission Electron Microscopy (TEM)

The Tecnai^TM^ (FEI, Hillsboro, OR, USA) TEM was used to confirm the mancozeb loading. Before analysis, samples were placed in a water bath sonicator. A drop of diluted conjugated nanoparticles was placed on a carbon-coated copper grid, followed by room-temperature air-drying. The photos were acquired using a 200 kV operational voltage using a facility from SAIF, AIIMS, New Delhi.

#### 2.4.4. Differential Scanning Calorimetry (DSC)

At LPU, Jalandhar’s CIF facility, a 3.0 mg sample was utilized to make thermographs using a DSC 4000 System (Perkin Elmer, Waltham, MA, USA) with a heating and cooling rate of 10 °C/min. Samples were heated from 30 to 445 °C. Pure nitrogen gas (99.99%) was pumped into the system at a rate of 20 mL/min.

### 2.5. In Vitro Study

#### 2.5.1. Encapsulation Efficiency (EE) and Loading Capacity (LC)

The supernatant was collected in a sterile tube after the nanocomposites were centrifuged at 15,000 rpm for 35 min. NanoDrop2000c (Thermo Scientific, Wilmington, DE, USA) was used to acquire mancozeb in the supernatant at 290 nm using the supernatant of their equal blank-attached nanoparticles as simple adjustments. The following equation was used to compute the encapsulation efficiency (EE%):

Total mancozeb added-mancozeb in supernatant/total mancozeb added ×100 = Encapsulation efficiency (percentage).

The following equation was used to compute the loading capacity (LC%):

(Mass of mancozeb in CSGA conjugated NPs)/(Weight of CSGA conjugated NPs recovered) × 100(1)

#### 2.5.2. Slow-Release Profile of Conjugated CSGA NPs

Mancozeb’s in vitro release from CSGA-conjugated nanoparticles was derived via dialysis tubing (Hi-Media Ltd., Mumbai). A total of 10 mg of the sonicated nanocomposite was added to 1.0 mL of sterile phosphate-buffered saline, PBS (pH 7.2). This was dished in a dialysis membrane with closed clips at one end and immersed in 10 mL of the same PBS in separate tiny beakers. At 37 °C, it was incubated in a shaker at 160 rpm; 1.0 mL of phosphate-buffered saline was pipetted out of each beaker after predetermined intervals of time (30 min), and the same volume of buffer was supplied to each beaker. The absorbance of the resultant solution was measured at 290 nm to estimate the quantity of mancozeb in the buffer using the standard curve.

#### 2.5.3. Antimicrobial Activity

The impact of NPs on the mycelial growth of a specific pathogen was established using the mycelium inhibition technique on potato dextrose agar (PDA, 2%) with certain modifications [23]. To make the final concentration of conjugated nanoparticles as 0.5, 1.0, and 1.5 ppm, autoclaved potato dextrose agar medium (Hi-Media, Mumbai, India; temp. 40 °C) was placed onto separate sterile Petri plates (90 mm × 15 mm) and allowed to harden. A 5.0 mm mycelial disc was removed from a seven-day-old test pathogen culture and placed in the center of the test Petri dish, where it was incubated at 28 ± 1 °C under constant monitoring. Growth was measured after four days for three replications, and the treated plates were compared to the control plates (without nanocomposites) to calculate the percent suppression of mycelia by NPs using an earlier approach [24].

dc − dt/dc 100 = percent inhibition(2)

where dc is the control mycelial diameter and dt is the mycelial treatment diameter.

### 2.6. In Vivo Study

#### 2.6.1. Bioefficacy in Pot House Conditions

To determine the bioefficacy of polymeric conjugated nanoparticles in controlling early blight and leaf spots in tomatoes (*Lycopersicon esculentum* L.), the study was conducted in pots filled with sandy soil in a glasshouse in natural light and temperature.

#### 2.6.2. Treatment of Seeds and Disease Detection

Seeds were properly rinsed and treated with 4% sodium hypochlorite for 10 min before being thoroughly cleaned. The seeds were dipped in CMC (carboxymethyl cellulose, 5.0 g in 100 mL DDW) for 10 min and air-dried. The seeds were then air-dried after being treated with conjugated nanoparticles (10 ppm) for 2 h and 30 min. Five tomato seeds per pot were planted in pots filled with soil (pH 7.7 at 20 °C) infected with pathogenic fungus [13]. The forty-day-old plants were sprayed with 15 mL of aqueous conidial solution (3.1 × 10^7^ CFU/mL) of specific pathogens and covered with clear plastic bags to maintain the humidity essential for disease outbreaks. Following the disease outbreak, a foliar spray of CSGA NPs (10 ppm and 15 mL/pot) was used to test the bioefficacy. As a positive control, commercial mancozeb was employed.

Disease severity (DS) was recorded randomly in the standard grade of 0–5 before the polymeric NP spray.


(3) $$\begin{array}{c} \text{Disease severity}\;(\%\mathrm{DS})\;=\\ \left(\mathrm{Sum}\;\mathrm{of}\;\mathrm{all}\;\mathrm{disease}\;\mathrm{ratings}\right)/\left(\mathrm{Total}\;\mathrm{plants}\;\mathrm{assessed}\;\times \;\mathrm{maximum}\;\mathrm{rating}\;\mathrm{scle}\right)\times 100 \end{array}$$

The disease control efficacy (% DCE) was calculated after the polymeric NP spray using the formula in [25].

For the overall health and vitality of the test plant, bioefficacy was measured using plant development characteristics, such as plant height, root–shoot ratio, and dry biomass. The dry weight of each tomato per plant was determined by placing the entire plant with roots in a brown envelope (3 plants per envelope) and drying it for seven days at 40 °C in a hot air oven.

### 2.7. Statistical Treatment of Data

All experiments were executed in triplicate, and the results are presented as mean ± standard deviation (SD). Statistical variances among sets were determined using one-way ANOVA. The statistical significance was accepted at a level of *p*-value ≤ 0.05 by a *t*-test. To handle statistical data, Microsoft Office Excel 2013 was utilized (Microsoft Corporation, Albuquerque, NM, USA).

## 3. Results and Discussion

### 3.1. Nanoparticle Size Optimization, Stability, and Physicochemical Characterization

**Figure 1:**
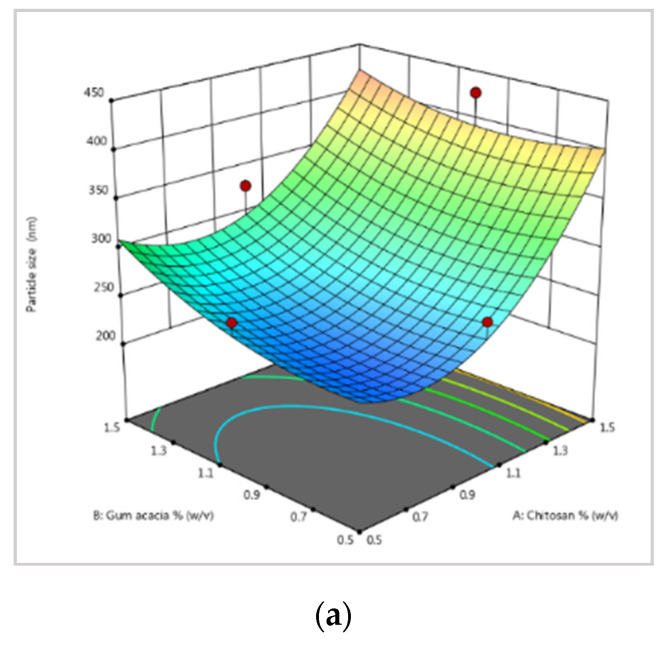

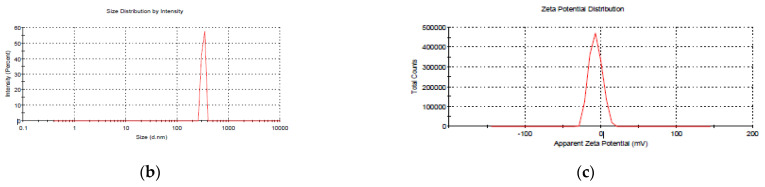
(**a**) Optimization of the concentration of gum acacia and chitosan for the particle size by response surface methodology (RSM); (**b**) mancozeb-loaded NP size; (**c**) mancozeb-loaded NP zeta potential.

The quantity of chitosan and gum acacia used in the experiment affected the particle sizes of the NPs in the initial trials. The optimization graph shows that the particle size increases with increasing chitosan and gum acacia concentrations (Figure 1a). In the case of chitosan, however, the impact is more pronounced.

The average diameter of blank NPs was 322.2 ± 0.9 nm, a 1.00 ± 0.1 PDI, and a zeta potential of −23.2 ± 0.08 mV. The size of NPs grew in response to increasing mancozeb concentrations (Table 1). 

The molecular weight, degree of deacetylation of the chitosan employed, the stirring speed, and time determine the size of the NPs [26]. A single strong peak at 403.7 ± 0.7 nm was detected for CSGA-1.0 (NPs containing 1.0 mg/mL mancozeb) with a zeta potential of −6.99 ± 0.5 mV (Figure 1b,c). Zeta potential values show the stability of NPs up to ±30 mV [27]. The nanocomposites formed were noticed in the synthesis mixture as a white, foggy haziness that settled at the bottom of the flask.

### 3.2. Storage Stability Determination

By preserving NPs in double-distilled water (DDW) at 4.0 °C for twenty days, the storage stability of the particles was established. The CSGA-1.0 size changed slightly from 403.7 ± 0.7 to 413.1 ± 0.8 nm (Table 1), which might be related to NP clumping since size differences in aggregated systems are common for various causes [28]. The zeta potential values obtained in this investigation ranged from +15.4 to +25.4 mV, indicating their stability.

The CSGA-1.0 formulation will be chosen for future research in vivo and in vitro because of its smaller size, higher loading capacity, and better storage stability. 

### 3.3. Ionic Group Interaction Study Using Fourier Transform Infrared Spectroscopy (FTIR)

**Figure 2:**
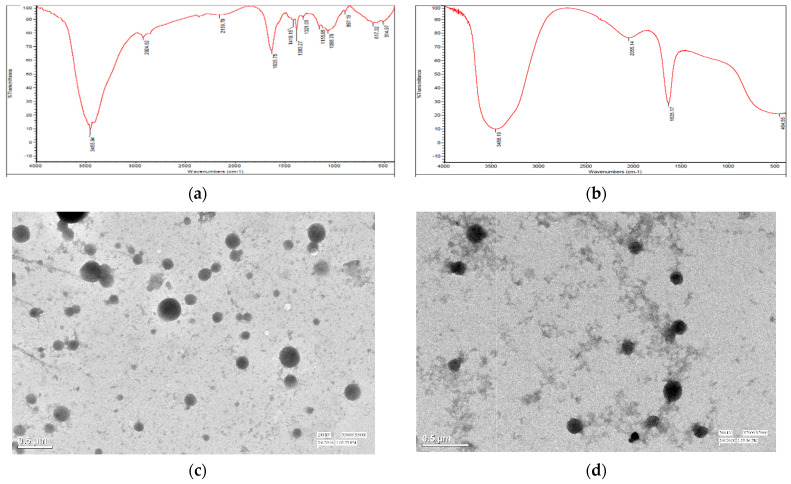
Fourier transform infrared spectra and TEM micrographs of conjugated nanoparticles; FTIR spectra of (**a**) gum acacia, (**b**) mancozeb-loaded NPs, (**c**) TEM micrograph (at 200 kV, ×5000 magnification, 0.5 µm scale bar) of blank NPs, (**d**) TEM of mancozeb-loaded NPs (at 200 kV, X7000 magnification, 0.5 µm scale).

Firm peaks indicate asymmetrical NH2 stretching vibration in CSGA-conjugated NPs at 3455.94 cm^−1^, whereas O-H stretching vibration is indicated by peaks at 3297.16 cm^−1^ (Figure 2a,b). In earlier research [29] with CSCRG NPs, it was found that a high peak at 3444.75 cm^−1^ in raw chitosan polymer suggested an asymmetrical NH_2_ stretching vibration [30]. Peaks in gum acacia powder indicated O-H stretching vibration at 3455.94 cm^−1^, C-H stretching vibration at 2929.66 cm^−1^, and o-amino- or o-hydroxyaryl ketones at 1635.25 cm^−1^ [31]. The peak at 1071.13 cm^−1^, on the other hand, indicated CH_3_ rocking vibration. Due to H-bonding between the COOH group of gum acacia and the NH_2_ of chitosan, the peak at 3400–3500 cm^−1^ seems to be wide. The fungicide loading within the nanocomposite is shown as a peak at 1635.17 cm^−1^ in mancozeb-loaded CSGA NPs. This is also supported by a previous study in which a researcher validated the encapsulation of acetamiprid in a nanoform by seeing a peak at 1633 cm^−1^, which corresponds to the stretching vibration of C=N/C-N [27].

### 3.4. Transmission Electron Microscopy (TEM)

TEM results (Figure 2c,d) confirmed the formation of discrete, small, round conjugated nanoparticles with fungicide seen inside the NPs as a dark gray spot for loaded NPs [32]. TEM micrographs verified crystalline nanocomposite formation and better understood the biopolymeric nanoparticle’s shape and size. The TEM pictures in this study show that the formed NPs were well dispersed, designating a monodispersed nature of synthesized nanoparticles and following previous research [27]. 

### 3.5. Differential Scanning Calorimetry (DSC)

**Figure 3:**
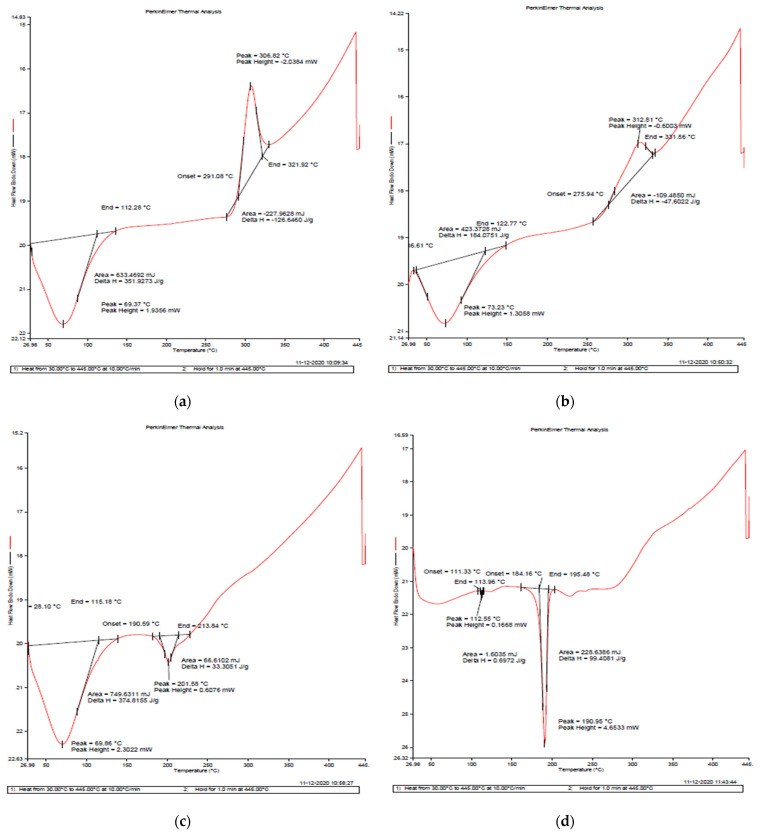

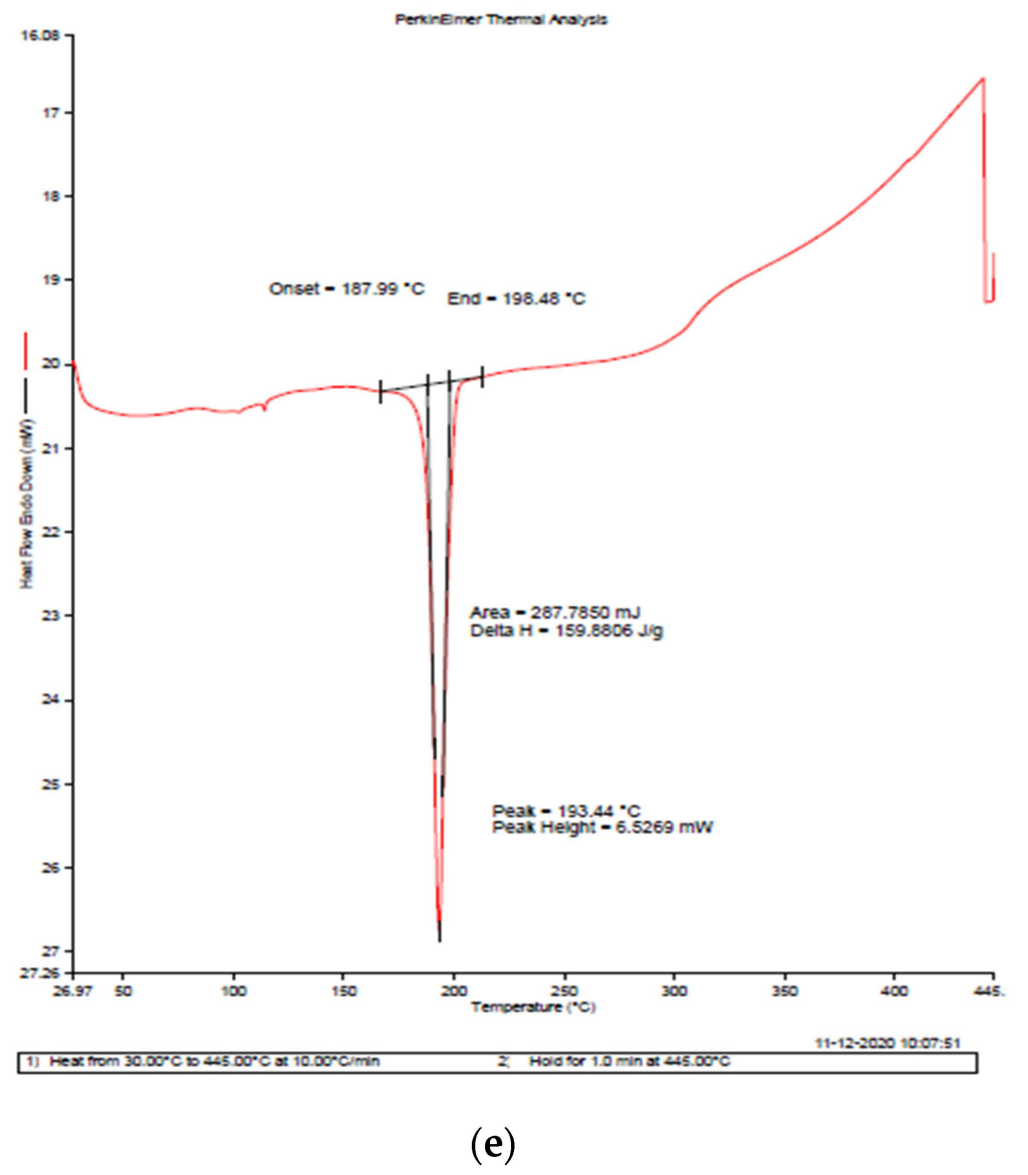
DSC thermograms of raw biopolymeric materials, (**a**) chitosan, (**b**) gum acacia and test samples, (**c**) CSGA blank, NPs (**d**) CSGA-1.0 NPs, (**e**) mancozeb.

The down peak in the graph represents an endothermic reaction, and the up peak represents an exothermic reaction (Figure 3). In mancozeb-loaded chitosan–gum acacia NPs, a sharp endothermic peak is observed at 190.95 °C with an enthalpy change (ΔH) of 99.406 J/g, which may be attributed to the melting of mancozeb [33]. Commercial mancozeb around 180 °C has a ΔH of 159.8806 J/g (Figure 3e). In contrast, blank and drug-loaded polymeric NPs have lower ΔH of 33.3051 J/g and 99.406 J/g, respectively (Figure 3c,d), which may be due to the glass transition of CSGA nanocomposites [34]. The lower ΔH, as compared to commercial mancozeb, makes both nanoformulations (blank and mancozeb-loaded NPs) more thermally stable than commercial mancozeb [29]. 

### 3.6. Encapsulation Efficiency and Loading Capacity

The encapsulation efficiency (EE) percentage and the loading capacity (LC) percentage of all three loaded nanoforms are shown in Table 2. A concentration-dependent pattern in the EE (%) and non-concentration-dependent increase in the percentage LC of mancozeb-loaded CSGA-conjugated nanoparticles was observed. Minimum and maximum EEs recorded were 15.95 ± 0.25 and 57.13 ± 0.29 for the formulations CSGA-0.5 and CSGA-1.5, respectively, with LC of 76.25 ± 0.26%, which might be due to excess mancozeb in the CSGA-1.5 sample [35]. Maximum LC (81.10 ± 0.18) was found for NPs containing 1.0 mg/mL mancozeb sample with optimum fungicide available for loading; results of the present study followed an earlier study [23,36].

### 3.7. Controlled Release Behavior

**Figure 4:**
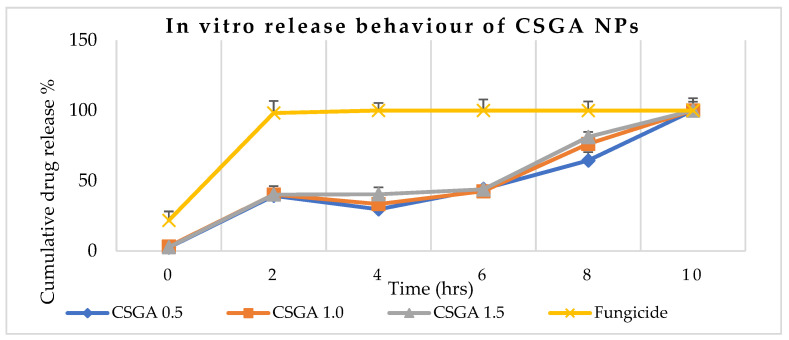
In vitro sustain release of mancozeb from chitosan–gum acacia-conjugated nanoparticles (CSGA). The yellow line represents commercial fungicide, while blue, saffron, and gray represent CSGA-0.5, 1.0, and 1.5, respectively.

The drug release from the conjugated nanoparticles was time-dependent and sustained-release owing to diffusion (Figure 4). The commercial fungicide released 100% within two hours, while NPs containing 0.5 mg/mL mancozeb (CSGA-0.5) had the best release mechanisms within 8 h, releasing 64% of total mancozeb content, while CSGA-1.5 formulation released 81% of total mancozeb content for the same time [37]. Because the active component of mancozeb is confined in the center of the polymer core, the release rate of nanoform is slower than that of commercial mancozeb [38,39]. Compared to commercial fungicides, this in vitro gradual release of NPs boosts plant absorption, reduces soil leaching, and reduces soil and water pollution. In a comparative investigation, the cumulative release percentage for carbendazim-loaded NPs was 50.4 ± 0.13 percent, compared to 67.7 ± 0.1% for pure carbendazim at pH 7.4 [40]. Another researcher [26] used an ionic gelation approach to collect marketable hexaconazole in chitosan NPs and obtained a sustained release of 99.91% over 86 h. There may be some chemical bonding between chitosan, gum, and fungicide as the polymers are oppositely charged, and fungicides also have both positive and negative charges, enhancing the release time. This enhanced interaction was also seen as a biocidal additive in paints and coatings [41].

### 3.8. In Vitro Antifungal Activity

**Figure 5:**
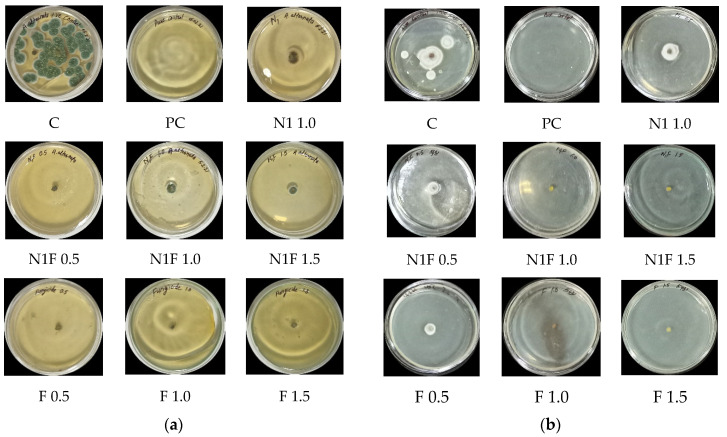
In vitro antifungal efficacy of blank- and fungicide- (1.0 mg/mL) loaded CSGA NPs at three concentrations (0.5, 1.0, and 1.5 ppm) using the mycelium inhibition method against tomato pathogens (**a**) *A. alternata*, (**b**) *S. lycopersici*; C—control, PC—pure control (Petri plates with PDA alone), N1—blank CSGA NPs, N1F—fungicide, F—fungicide.

Maximum inhibition (85.2 ± 0.7%) was seen in the case of *Alternaria alternata* in a mancozeb-loaded formulation at 0.5 ppm and is almost the same as commercial mancozeb at 1.5 ppm (Figure 5a), which might be attributed to the formulation’s increased zeta potential. Mancozeb-loaded CSGA NPs had the best antimicrobial efficacy against *S. lycopersici* at 1.0 and 1.5 ppm (100% inhibition at both concentrations). These findings matched prior research in which carbendazim-loaded polymeric nanoparticles were evaluated against *F. oxysporum* and *A. parasiticus* and showed 100% fungal suppression at 0.5 and 1.0 ppm [23]. In contrast, it exhibited 62.1 ± 0.7% inhibition at 0.5 ppm, equivalent to commercial mancozeb (56.1 ± 0.7%). Table 3 and Figure 5a,b) indicate the percentage inhibition shown by NPs.

Nanofungicides have better efficiency than traditional fungicides owing to the formation of reactive oxygen species (ROS), membrane permeability due to their tiny size, and enzyme-related defense responses in plants against fungi [22,42]. Another study obtained more substantial repellents (around 80%) against two key agricultural pests (whitefly (*Bemisia tabaci*) and spider mites) while working with a hydrogel containing botanical repellents encapsulated in zein nanoparticles [43]. In previous research, *Rhizoctonia* sp. and *Alternaria* sp. strains were shown to be the most susceptible to chitosan–zinc nanocomposites, followed by chitosan–copper nanocomposites [24].

### 3.9. In Vivo Antifungal Efficacy

The control plants exhibited a disease severity (DS) of 42.9 ± 3.3% for early blight and 40.9 ± 0.8% for leaf spot disease after seven days of conidial spraying of a pathogenic fungus, as shown in Table 4. Disease control efficiency (DCE, in percent) in pathogen-treated plants for loaded NPs was found to be 66.2 ± 5.0% and 70.7 ± 1.6%, respectively, against early blight and leaf spot diseases, which is comparable to sick pathogen plants treated with commercial fungicide (66.0 ± 3.5% and 68.5 ± 1.1%) (Table 4). Similar to metolachlor alone, polymeric (PEG-PLGA) nanoparticles loaded with the weed killer metolachlor showed strong herbicidal action against *Digitaria sanguinalis*, *Oryza sativa,* and *Arabidopsis thaliana* in the research [44].

In the pot settings, chitosan nanoparticles, and chitosan–silver nanocomposites were as efficient as traditional fungicides, such as copper oxychloride 525]. Another study used silver nanoparticles produced by rhizospheric chickpea microorganisms to treat chickpea wilt illness in vivo (*Cicer arietinum*). The current investigation of disease inhibition is in line with earlier research [15]. Earlier, applying chitosan hexaconazole–dazomet to treat *G. boninense*-caused basal stem rot disease in palm trees reduced the disease by 74.5% [45]. These findings corroborate the validity of the current research.

### 3.10. Plant Growth Parameters Study

For this study, three parameters, namely germination percentage, dry mass per plant, and root-shoot ratio, were chosen for the pot house conditions.

#### 3.10.1. Effect of Treatment on Germination Percentage 

The tomato is an important crop sown globally. No comprehensive research data are available on the effects of chitosan–gum acacia nanoparticles on plant germination indices in pot or field conditions. The lowest germination (60%, in both) was observed in plants treated with fungicide alone (F) and plants treated with blank NPs, which were made sick with the pathogen *A. alternata* (N1P1). The highest germination (78%) was recorded in plants treated with *S. lycopersici* and loaded NPs (N1FP2). It was pretty high as compared to plants treated with commercial mancozeb alone (F-60%), plants treated with pathogen *S. lycopersici* alone (CP2-72%), and fungicide-treated plants, which were made sick with *S. lycopersici* (FP2-62%). From the above data, it can be conferred that the loaded NPs (N1FP2) treated with *S. lycopersici* showed good germination (78%) compared to commercial mancozeb (60%). The same pattern of germination in the present study was found in earlier research, where the germination of the seeds treated with nanoformulation was 96%, while pure carbendazim showed decreased (60%) germination [23].

#### 3.10.2. Tomato Dry Mass per Plant

The highest mass of 1328.3 mg was obtained for plants treated with fungicide-loaded NPs, and made sick with *S. lycopersici* (N1FP2), followed by 1102.5 mg for fungicide-treated plants made sick with *S. lycopersici* (FP2). Plants treated with fungicide alone (F) had a mass of 630 mg, which is relatively lower than all the NP-treated plants. Hence, all treatments, except N1P1 (plants treated with blank NPs, and made sick with *A. alternata*), had a positive effect on the plant biomass or weight per plant compared to the fungicide alone at a concentration of 10 ppm of foliar spray of NPs with 1.0 mg/mL of mancozeb, i.e., CSGA-1.0. In an earlier study, high concentrations of ZnO and silver NPs harshly affected the growth of tomato and wheat plants, respectively [46,47]. Earlier research also found that root and shoot growths in tomatoes were more affected by silver usage than seed germination [48].

#### 3.10.3. Root–Shoot Ratio of Plants

The most significant shoot lengths of 19.5 and 19 cm were observed in plants treated with mancozeb-loaded nanocomposites infected with *A. alternata* and *S. lycopersici,* respectively. There may be some interactions between mancozeb-loaded NPs and pathogens, which caused an increase in shoot length. In a study, CeO_2_ NPs were found in soybean roots and root nodules after growth in soil treated with NPs [49]. A similar transfer in corn plants was found, and CeO_2_ NPs around vascular vessels supported that the particles found their way to the transportation system and moved through the xylem compelled by transpiration [50]. The most miniature shoots (11 cm) were seen in control plants, commercial fungicide-treated plants, followed by plants treated with blank and loaded nanocomposites (11.5 cm). The most significant root length (15.3 cm) was found in plants treated with commercial mancozeb and infected with *A. alternata* while the smallest root length of 8.0 cm was observed in control plants. So it can be concluded that nanocomposites positively affected the shoot and root lengths of plants compared to non-treated plants.

## 4. Conclusions

Conventional mancozeb has metals, such as zinc and manganese, which interfere with the living entity and cause significant environmental, soil, and health problems because of leaching via soil and water. Considering the hazards of commercial mancozeb, the biopolymeric nanocomposites in the current study showed a good EE and LC by sandwiching mancozeb in oppositely-charged biopolymers. Dialysis tubing showed slow and sustained release of mancozeb from encapsulated nanocomposite, which may be the reason for higher or comparable DCE percentages against *A. alternata* and *S. lycopersici* compared to commercial mancozeb, and can be used for environmental protection. Thus, these nanoformulations may be explored for the site-directed delivery of mancozeb for disease control in plants with minimalistic fungicide loaded to minimize environmental, soil, and human health problems.

## Figures and Tables

**Table 1:** CSGA NP size, PDI, and zeta potential at native pH; freshly prepared, and their storage stability after 20 days in DDW at 4 °C.

CSGA NPs	Size (nm)	PDI	Zeta Potential (mV)
Freshly prepared nanoparticles			
CSGA Blank	322.2 ± 0.9	1.00 ± 0.1	−23.2 ± 0.08
CSGA-1.0	403.7 ± 0.7	0.789 ± 0.1	−6.99 ± 0.5
Storage stability of conjugated nanoparticles at 4 °C			
CSGA Blank	343.9 ± 0.5	0.255 ± 0.3	21.3 ± 0.4
CSGA-1.0	413.1 ± 0.8	0.301 ± 0.7	25.0 ± 0.3

Mean ± standard deviation in replication of three.

**Table 2:** Mancozeb encapsulation and loading capacity of CSGA NPs.

Formulation	Encapsulation Efficiency (%)	Loading Capacity (%)
CSGA-0.5	15.95 ± 0.25	47.57 ± 0.39
CSGA-1.0	36.62 ± 0.31	81.10 ± 0.18
CSGA-1.5	57.13 ± 0.29	76.25 ± 0.26

Mean ± standard deviation in replication of three.

**Table 3:** In vitro antifungal efficacy of mancozeb-loaded (1.0 mg/mL) CSGA-1.0 nanocomposites.

Fungi	Nanoformulation with Mancozeb (ppm)	CSGA NPs	NPs % Inhibition = dc − dt/dc × 100	Mancozeb (ppm)	Mancozeb	Mancozeb % Inhibition = dc − dt/dc × 100
		Fungi Diameter (mm)			Fungi Diameter (mm)	
*A. alternata* (ITCC6343)	Blank NPs, N 1.0	18.5 ± 0.45	76.1 ± 0.22 b		-	-
	Loaded NPs, NF 0.5	11.5 ± 0.23	85.2 ± 0.61 b	F 0.5	12.0 ± 1.4	84.5 ± 1.4 b
	Loaded NPs, NF 1.0	15.5 ± 0.71	80.0 ± 0.42 b	F 1.0	11.5 ± 0.7	85.2 ± 0.7 b
	Loaded NPs, NF1.5	12.5 ± 0.96	83.9 ± 0.54 b	F 1.5	10.5 ± 0.7	86.5 ± 0.7 b
*S. lycopersici* (ITCC5431)	Blank NPs, N 1.0	15.5 ± 0.7	53.0 ± 0.7		-	-
	Loaded NPs, NF 0.5	12.5 ± 0.7	62.1 ± 0.7	F 0.5	14.5 ± 0.7	56.1 ± 0.7 c
	Loaded NPs, NF 1.0	0	100 ± 0 a	F 1.0	0	100 ± 0 a
	Loaded NPs, NF 1.5	0	100 ± 0 a	F 1.5	0	100 ± 0 a

Each value is a mean of a triplicate. Mean ± standard deviation followed by the same letter in the treatment column indicate that values are not significantly different at *p* ≤ 0.05, as determined by a *t*-test.

**Table 4:** Treatment effects of CSGA NPs on percentage disease severity (DS) and disease control efficacy (DCE) in tomato plants under pot house conditions against early blight and leaf spots.

Treatment	*A. alternata*		*S. lycopersici*	
	% DS	% DCE	% DS	% DCE
Pure control, C	16.1 ± 1.4	00.0 ± 0.0	12.7 ± 1.5	00.0 ± 0.0
Control + Pathogen, CP	42.9 ± 3.3	00.0 ± 0.0	40.9 ± 0.8	00.0 ± 0.0
Fungicide, F	10.1 ± 1.9	76.5 ± 5.8 a	10.2 ± 1.8	75.1 ± 1.8 a
Fungicide + Pathogen, FP	14.6 ± 3.4	66.0 ± 3.5 b	12.9 ± 2.3	68.5 ± 1.1 b
Blank NPs, N1	12.9 ± 0.5	69.9 ± 3.7 c	16.0 ± 1.7	60.9 ± 1.9 c
Blank NPs + Pathogen, N1P	15.6 ± 3.4	63.6 ± 1.5	15.0 ± 2.8	63.3 ± 4.6
Loaded NPs, N1F	10.0 ± 1.2	76.7 ± 3.4 a	10.5 ± 1.2	77.3 ± 1.6 a
Loaded NPs + Pathogen, N1FP	14.5 ± 1.4	66.2 ± 5.0 a	12.0 ± 2.9	70.7 ± 1.6 a

Each value is a mean of a triplicate. Mean ± standard deviation followed by the same letter in the treatment column indicate that values are not significantly different at *p* ≤ 0.05, as determined by a *t*-test.

## Data Availability

Data will be made available upon request.
